# Not all cows are epidemiologically equal: quantifying the risks of bovine viral diarrhoea virus (BVDV) transmission through cattle movements

**DOI:** 10.1186/s13567-014-0110-y

**Published:** 2014-10-17

**Authors:** M Carolyn Gates, Roger W Humphry, George J Gunn, Mark E J Woolhouse

**Affiliations:** Epidemiology Group, Centre for Immunity, Infection and Evolution, School of Biological Sciences, University of Edinburgh, Ashworth Laboratories, Kings Buildings, West Mains Road, Edinburgh, EH9 3JT, UK; Epidemiology Research Unit, SRUC, Drummondhill, Stratherrick Road, Inverness, IV2 4JZ, UK

## Abstract

Many economically important cattle diseases spread between herds through livestock movements. Traditionally, most transmission models have assumed that all purchased cattle carry the same risk of generating outbreaks in the destination herd. Using data on bovine viral diarrhoea virus (BVDV) in Scotland as a case example, this study provides empirical and theoretical evidence that the risk of disease transmission varies substantially based on the animal and herd demographic characteristics at the time of purchase. Multivariable logistic regression analysis revealed that purchasing pregnant heifers and open cows sold with a calf at foot were associated with an increased risk of beef herds being seropositive for BVDV. Based on the results from a dynamic within-herd simulation model, these findings may be partly explained by the age-related probability of animals being persistently infected with BVDV as well as the herd demographic structure at the time of animal introductions. There was also evidence that an epidemiologically important network statistic, “betweenness centrality” (a measure frequently associated with the potential for herds to acquire and transmit disease), was significantly higher for herds that supplied these particular types of replacement beef cattle. The trends for dairy herds were not as clear, although there was some evidence that open heifers and open lactating cows were associated with an increased risk of BVDV. Overall, these findings have important implications for developing simulation models that more accurately reflect the industry-level transmission dynamics of infectious cattle diseases.

## Introduction

### Background

Infectious diseases cause significant financial losses for the cattle industry through their detrimental effects on animal health and performance [1]. As such, researchers are continually developing more sophisticated epidemiological models to better understand how disease control resources can be applied more cost-effectively across the large population of cattle herds [2-4]. Cattle movements have received particular attention in recent years both because of their central role in the epidemiology of many economically important cattle diseases [5-7] and because the movements of individual cattle have been explicitly recorded in databases across the European Union since 1998 [8]. The latter has provided researchers with an unprecedented opportunity to study the dynamics of directly transmissible infectious diseases. Using network analysis based approaches, it has been consistently shown that targeting control measures at the small number of herds or movements that are highly connected in the trade network can lead to significantly greater reductions in disease prevalence than targeting the same number of herds or movements at random [9-12].

From a practical perspective, these findings must be interpreted with some caution as most models assume that purchased cattle all carry the same risk of generating disease outbreaks in the destination herd. As numerous empirical studies have shown, the probability of any individual animal being infected or transmitting disease to susceptible cattle is strongly influenced by factors such as age, production type, and on-farm management practices [13-16]. For example, contagious mastitis pathogens are highly unlikely to spread through the movements of male cattle or store calves purchased for fattening, whereas older lactating dams are predicted to have a significantly increased risk based on the higher prevalence of disease and greater opportunity to spread disease through contaminated milking equipment [17,18]. Identifying cattle movements that are associated with the greatest risk of infectious disease transmission has important implications for refining future epidemiological models and disease control strategies. In this analysis, we use data on bovine viral diarrhoea virus (BVDV) in Scotland as a case example to illustrate that not all cows in the movement network are epidemiologically equal.

### BVDV epidemiology

BVDV is an economically important pathogen for the cattle industry due to its negative effects on herd reproduction and calf performance [19-21]. During acute outbreaks, cattle infected with BVDV may exhibit non-specific clinical signs of depression, inappetence, fever, and diarrhoea leading to transient declines in milk production, growth performance, and animal fertility [22]. More serious complications arise when BVDV crosses the placental barrier in pregnant cattle. Foetal infections have been associated with early embryonic death, abortions, stillbirths, congenital abnormalities, and the development of persistent infections in calves that gain immunotolerance to BVDV through vertical transmission of the virus during early gestation [23]. Persistently infected (PI) calves shed large quantities of virus for life and are primarily responsible for sustaining disease transmission at the population level [24]. Due to underlying immunosuppression and the development of fatal mucosal disease, few PI cattle survive beyond three years of age [25,26]. However, those that appear clinically normal are at risk of being sold to other herds as store cattle or breeding replacements leading to the exposure of pregnant dams in the gestational risk period for generating additional PI calves [27-29].

Given the importance of BVDV, many statistical and epidemiological models have been developed to identify risk factors for BVDV transmission and opportunities for controlling disease more cost-effectively through targeted interventions [2,13,30-32]. While most published studies agree that maintaining an open breeding herd is the primary risk factor for disease introductions [31-35], there are likely specific cohorts of purchased cattle that are at increased risk of generating outbreaks in the destination herd. For example, the probability that any given batch of purchased cattle will contain at least one PI animal is expected to decrease with age given the higher observed mortality rates amongst PI calves [25,36]. However, older animals purchased as replacement breeding cattle have a greater probability of being directly co-mingled with susceptible breeding dams than animals purchased as yearling heifers. Seropositive dams that are pregnant at the time of purchase carry the additional risk of introducing BVDV through the birth of PI calves [37], particularly since there are few reliable prenatal tests to determine the BVDV status of the foetus [38-40].

### Objectives

Our study uses three independent analytical approaches to determine the risk associated with particular types of purchased replacement breeding cattle. First, a traditional risk factor analysis is performed using serological data from beef and dairy herds that were surveyed as part of national seroprevalence studies in Scotland from 2006 to 2008 [41,42]. Second, a dynamic within-herd simulation model is used to determine whether the observed trends could be explained by the known epidemiological features of BVDV infections in cattle as well as the demographic structure of Scottish herds at the time when the different types of purchased replacement breeding cattle are typically introduced. Third, the network betweenness centrality score (a frequently used measure of the potential for herds to acquire and transmit disease through movement networks) was calculated for each source herd that supplied replacement breeding cattle to determine whether contact network structure also contributed to the differential risk associated with the different types of replacement breeding cattle movements.

## Materials and methods

### Cattle movement data

The Cattle Tracing System (CTS) database contains virtually complete records of the births, deaths, and movements of individual cattle in Great Britain since 2001. Each movement record includes basic information on the animal identification number, departure location, destination location, and movement date that may be linked with other recorded animal demographic information in the CTS database (age, sex, breed, and recorded calvings) to infer the animal’s production purpose at the time of movement. These records may also be used to generate an inventory of individual cattle present on a given farm at any given time point. This analysis focused on the movements of replacement breeding cattle since these are a well-established risk factor for BVDV transmission [22]. Data from the beef and dairy industries were analysed separately throughout the study due to inherent differences in herd management practices and demographic structure.

An animal was classified as a purchased replacement breeding heifer (1) if the sex was registered as female, (2) if there were no recorded calvings prior to the movement, (3) if the animal was born on a different location than the destination farm, and (4) if the next recorded calving after the movement took place on the destination farm or, if there were no recorded calvings after the movement, that the animal survived beyond 30 months of age. It was assumed that animals intended for human consumption rather than breeding would be slaughtered by 30 months of age to comply with bovine spongiform encephalopathy (BSE) regulations in the United Kingdom [43]. An animal was classified as a replacement breeding cow (1) if the sex was registered as female, (2) if there was at least one recorded calving that took place prior to the movement, (3) if the most recent calving prior to the movement took place on a different farm than the destination farm, and (4) if the next recorded calving after the movement took place on the destination farm.

The replacement breeding cattle groups were further subdivided by pregnancy and lactation status to generate six total production groups: open heifers, pregnant heifers, open dry cows, open lactating cows, pregnant dry cows, and pregnant lactating cows. Animals that were fewer than 280 days from calving at the time of movement were considered pregnant, while animals that were greater than or equal to 280 days from calving were considered open. Beef breed cows that were moved onto the farm at the same time as their calf were considered to be in lactation with a calf at foot, while those moved without a calf were considered to be dry cows. Dairy breed cows that were moved onto the farm within 305 days of the previous calving date were also considered to be in lactation, while those moved greater than 305 days post-calving were considered to be dry cows.

### Empirical risk factor analysis

#### Serological data

A survey of 301 randomly selected beef herds was performed in Scotland between October 2006 and September 2007 to estimate the national herd-level prevalence of BVDV [42]. During the farm visit, blood samples were obtained from 10 randomly selected animals between 6 and 16 months of age (for 27 of the herds the number of animals sampled differed from 10, typically due to the group size being too small). The blood samples were processed using an indirect BVDV antibody ELISA to obtain antibody titres and were classified as positive or negative based on the percentage positivity (PP) score. Based on the two higher mixture distributions described previously for these data [42], the 225 herds with less than 26.3% prevalence amongst young stock were considered control herds, and the 76 herds with a within-group prevalence of more than or equal to 26.3%, were considered seropositive case herds.

A survey of 374 dairy herds was also performed in Scotland between October 2007 and May 2008 to estimate the prevalence of antibodies to BVDV in bulk tank milk samples [41]. The bulk milk tank samples were obtained through the farm’s milk purchaser at the time of collection and processed using indirect BVDV antibody ELISA to obtain the percentage positivity (PP) score. The 154 herds that reported vaccinating cattle for BVDV were excluded from the analysis since it was not possible to determine whether the antibodies present in the bulk milk tank sample were due to the vaccine or due to natural infection. Following the Swedish BVDV eradication class system, the remaining 220 herds that did not vaccinate for BVDV were assigned into one of four groups based on their PP score. Class 0 herds were considered unlikely to have any seropositive animals indicating a low probability of BVDV infection, while Class 3 herds were considered highly likely to have many seropositive animals indicating a recent or active infection. For the purpose of this analysis, the 77 herds designated as Class 0 or Class 1 were considered control herds and the remaining 143 herds designated as Class 2 or Class 3 were considered seropositive case herds.

The questionnaire returned by the surveyed beef and dairy farmers identified farms through the main postal address and so to link the serological results with records from the CTS database, attempts were made to match the farm address against a database of CPH codes provided by the Scottish government. Farms for which there was no available CPH code and farms for which there was an obvious discrepancy between the questionnaire estimates of herd size and CTS database estimates of herd size were excluded from the analysis. These discrepancies were most likely due to cattle being housed on a different location than the main farm address recorded in the survey and resulted in the loss of 46 beef herds (17% of the original 301 herds) and 31 dairy herds (14% of the 220 non-vaccinating herds). The final sample therefore contained 255 beef herds (67 case herds and 188 control herds) and 189 dairy herds (122 case herds and 67 control herds).

#### Statistical analysis

Data from the remaining 255 beef herds and 189 dairy herds were used to explore the relative risk of purchased replacement breeding cattle causing outbreaks in the destination herd. For each herd, the total number of cattle purchased in the two year period prior to serological sampling was recorded. The two year time window was selected because of the uncertainty in when BVDV may have been introduced to the herd. A series of six binary categorical variables were created representing each of replacement breeding cattle types (open heifer, pregnant heifer, open cow (dry), open cow (lactating), pregnant cow (dry) and pregnant cow (lactating)). The levels of the variables were “None purchased” and “At least one purchased”. Herds that purchased no cattle in the 2 year period prior to sampling were considered closed herds. The odds of a closed herd being seropositive for BVDV were initially calculated and the remaining analyses then focused on the subset of 233 open beef herds and 150 open dairy herds.

Preliminary univariate screens were performed to select variables for inclusion in the final multivariate logistic regression models. All six movement variables were associated with BVDV seropositivity at a *p*-value < 0.20 and were therefore retained. Thereafter, components of the final multivariate models were determined by a backwards stepwise elimination process in which variables with the highest *p*-values were sequentially dropped from the model until all the remaining variables had a *p*-value < 0.05. Forwards stepwise selection was then performed adding in each of the eliminated variables in turn and checking for *p*-values of < 0.05 to ensure that no variables were excluded based on the order of elimination.

### Within-herd simulation model

A stochastic individual-based within-herd simulation model was then developed to determine whether the general trends in the empirical risk associated with different types of replacement breeding cattle movements could be explained by the combination of (1) the theoretical risk of the animal being infected with BVDV or carrying an infected calf at the time of purchase, which is a function of the animal’s age and pregnancy status as well as the expected disease prevalence and (2) the theoretical risk of disease subsequently spreading to at least one susceptible dam during the gestational risk period for generating PI calves, which is a function of the herd demographic structure at the time of purchase as well as the ability for disease to spread within and between management subgroups.

This analysis focused on the subset of 2895 beef herds and 546 dairy herds in Scotland with exclusively beef or dairy calvings (1) that housed cattle continuously over the period from July 2004 through June 2007 to ensure that sufficient post-hoc data was available to classify animals into production groups, (2) that purchased at least one replacement breeding animal in the period from July 2004 through June 2005 since the focus of the analysis was on BVDV introductions through replacement breeding cattle movements, and (3) that had at least 20 recorded calvings per year to rule out hobby farms and farms that ceased production during the study period. The within-herd model included a demographic component to capture the typical management subgroups of Scottish beef and dairy herds, a seeding component to describe the introduction of BVDV through one of the purchased replacement breeding cattle, and a disease component to describe the subsequent transmission of BVDV within and between management subgroups after the animal was introduced. The distribution of animals across the different management subgroups within each herd and the movements on and off the farm were modelled directly from CTS records to account for real world heterogeneity in herd demographic structure. The simulation model was implemented in the C programming language.

#### Demographic component

**Figure 1:**
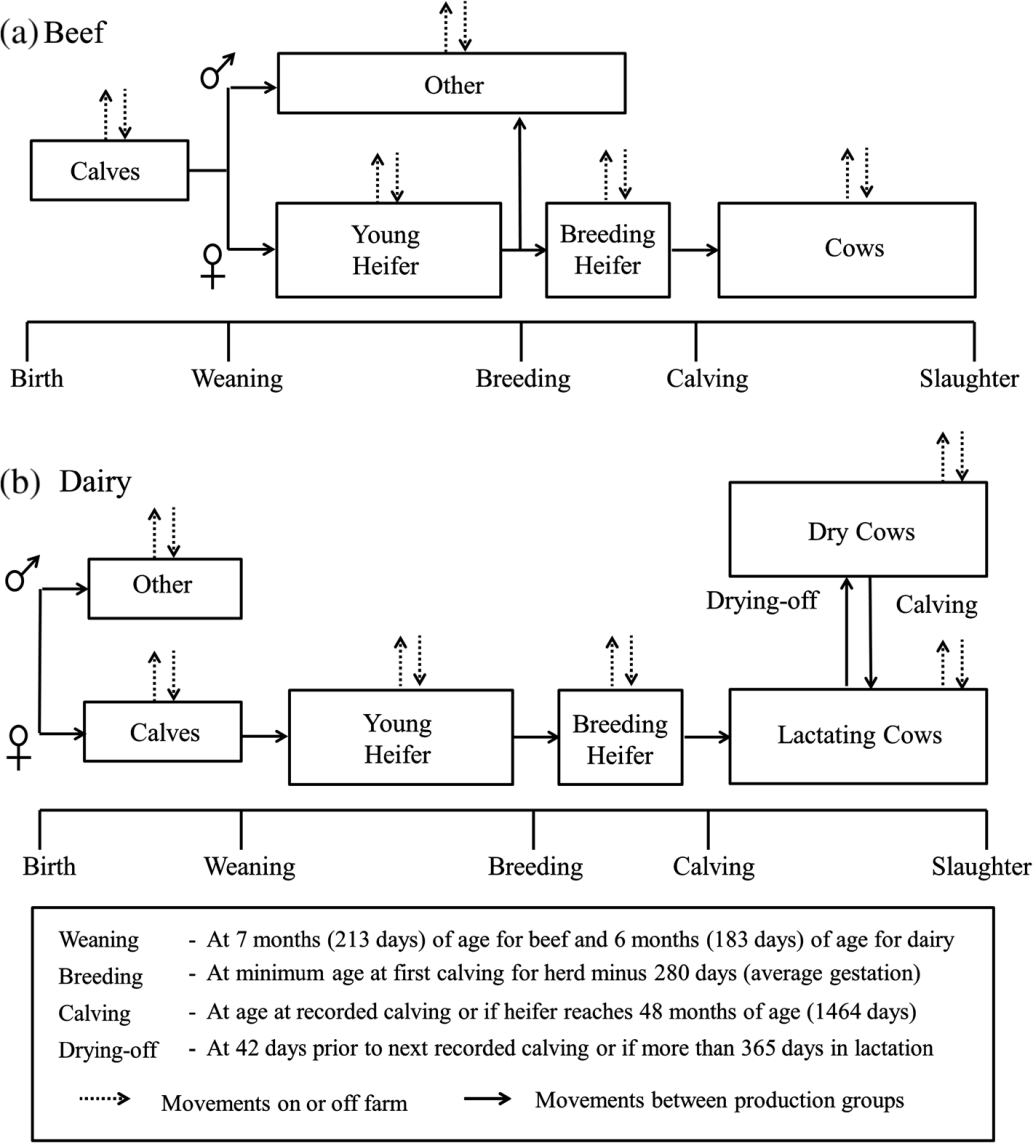
**Diagrammatic representation of the production subgroups in typical Scottish (a) beef and (b) dairy herds.** This informs the demographic component of the within-herd BVDV simulation model.

Records for all cattle present in the study herds from 01 July 2004 through 01 July 2007 were extracted from the CTS database. Each animal was initialized as a virtual object that carried information on its age, production subgroup, pregnancy status, and disease status at any given time point. Based on expert opinion from farmers and veterinarians on the typical management structure of Scottish beef and dairy herds, animals were assigned into one of the production subgroups shown in Figure 1.

In beef herds, all calves born in the herd remained with their dams until a fixed weaning age of 213 days (7 months). At weaning, male calves were transferred into the “other” group and female calves were transferred into the “young heifer” group. At the minimum age at first breeding for the farm, heifers that subsequently delivered a calf or survived beyond 30 months of age were transferred into the “breeding heifer” group. All other heifers were assumed to be intended for fattening and transferred into the “other” group. The minimum age at first breeding was calculated by taking the minimum age at first calving for heifers over the three year study period and subtracting the average gestation length of 280 days. Heifers remained in the “breeding heifer” group until calving or until reaching 48 months of age. Given limitations in the CTS data, it was not possible to further separate animals into spring and fall calving units on farms with year-round calving patterns or to identify exposure to male cattle kept or purchased as breeding bulls.

In dairy herds, calves were removed from their dams immediately at birth. All male calves and crossbreed calves (defined as beef breed animals born to dairy breed dams) were transferred into the “other” group. Female calves remained in the “calf” group until a fixed age of 183 days (6 months) and were then transferred into the ‘young heifer’ group until the minimum age at first breeding for the farm. Heifers remained in the “breeding heifer” group until calving or until reaching 48 months of age. After calving, the dams were transferred to the “lactating cow” subgroup until 42 days prior to the next calving, reflecting the average dry period for dairy cattle, or until more than 365 days into lactation if the animal failed to conceive. Animals remained in the “dry cow” subgroup until the next recorded calving date or movement off the farm. An average gestation length of 280 days was again assumed.

On each day of the simulation, the herd demographic structure was updated in four steps: (1) animals within the herd were transitioned between production subgroups as appropriate, (2) animals were removed from the herd based on an event list of deaths and off-movements, (3) the pregnancy status of animals was updated based on an event list of breeding dates derived by subtracting 280 days from the next recorded calving date, and (4) animals were added to the herd based on an event list of births and on-movements.

#### Seeding component

Each disease simulation-run for an individual herd began on 01 July 2004. All animals in the herd were initially assumed to be susceptible to BVDV to represent a true outbreak scenario. In each simulation run, only one of the replacement breeding cattle movements occurring from 01 July 2004 through 30 June 2005 was selected as the potential disease “seeding event” and all other cattle moved onto the farm before or after this movement were assumed to be susceptible to BVDV (i.e. a single introduction of BVDV with no re-introduction through movements or local spread). Since it was not possible to determine the true BVDV status of the source herds at the time of movement, it was assumed that all source herds were potentially infected with BVDV. The objective was not to make inferences about the true within-herd and between-herd dynamics of BVDV outbreaks, but rather to provide a simple framework for comparing the relative risk associated with the different types of replacement breeding cattle movements and the demographic structure of the herds at the time when replacement breeding cattle were introduced. Over the one year period from 01 July 2004 through 30 June 2005, the beef study herds collectively purchased 44 485 replacement breeding cattle and the dairy study herds collectively purchased 10 023 replacement breeding cattle.

**Figure 2:**
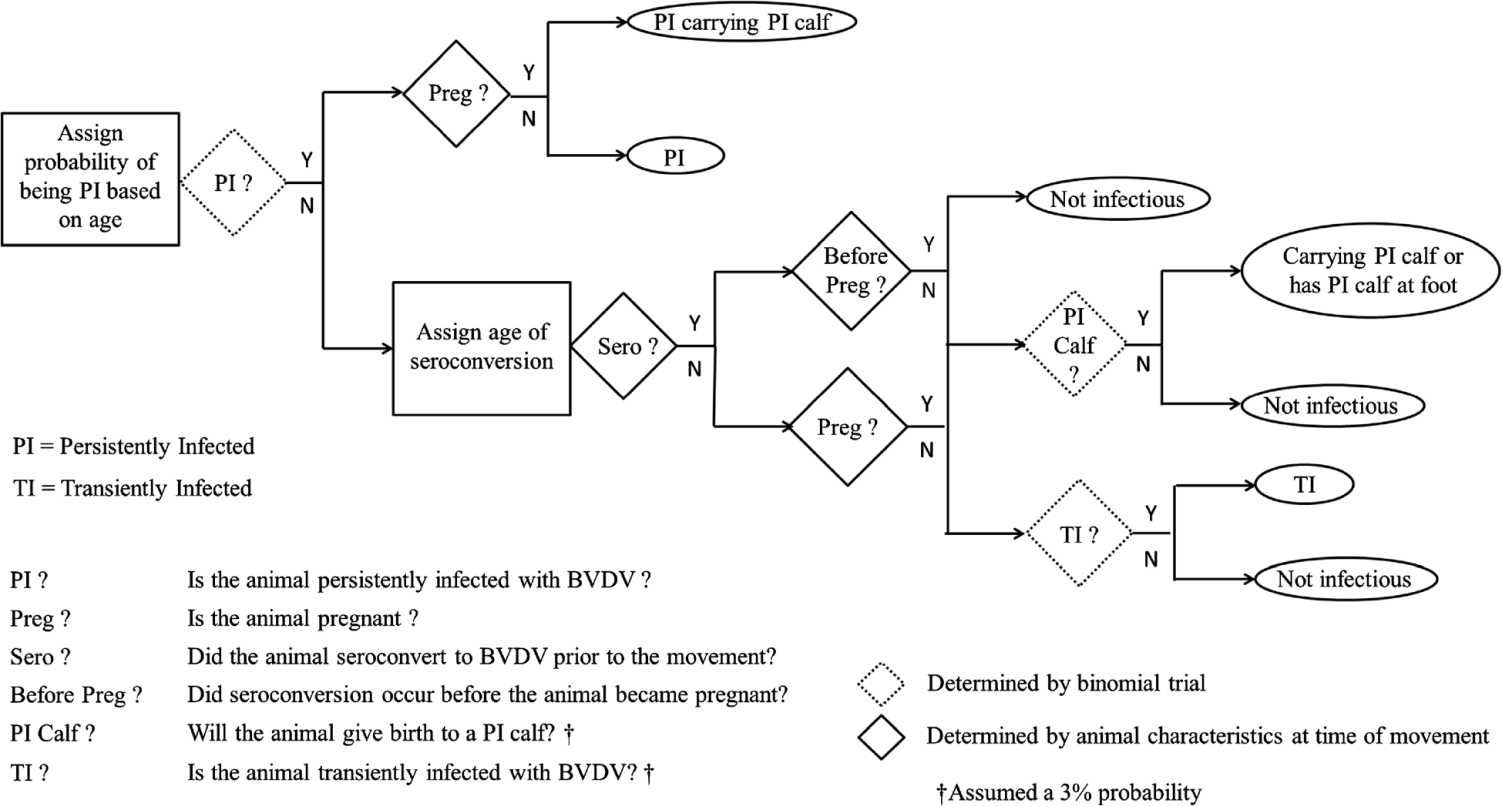
**Stochastic decision tree to determine the BVDV status of purchased replacement breeding cattle.** This informs the seeding component of the within-herd BVDV simulation model.

The infection status of each replacement breeding animal was determined stochastically at the start of each simulation according to the decision tree process presented in Figure 2. First, each animal was assigned a probability of being PI (ρ_pi_) based on its age (Equation 1). It was assumed that the average prevalence of PI cattle at birth (ρ_0_) in infected herds was 3% and the probability of being PI decayed as an exponential function of age with a half-life (*t*_*1*/*2*_) of 365 days to reflect the decreased survival rate of PI calves [44-48].

1 $$ {\rho}_{pi} = {\rho}_0\ast {e}^{Age\ *\ \frac{- \ln (2)}{t_{1/2}}} $$

Where ρ_0_ is equal to the average prevalence of PI cattle at birth, *Age* is equal to the animal’s age at the time of purchase in days, and *t*_*1*/*2*_ is equal to the half-life of PI cattle.

A binomial trial was then used to determine whether or not the replacement animal was PI. If the replacement animal was not PI, it was then assigned a random age of seroconversion (*Age*_*sero*_) sampled from an exponential distribution with a half-life of 1095 days (Equation 2) to reflect the average age of seroconversion in the general population of cattle [35,49,50]. The purpose was to account for the increasing probability that older cattle will have been previously exposed to BVDV either through natural infection or immunization.

2 $$ Ag{e}_{sero}= \ln \left(1-u\right)/\ \left(\ \frac{- \ln (2)}{t_{\frac{1}{2}}}\ \right) $$

Where *u* is equal to a uniform random number between 0 and 1and *t*_*1*/*2*_ is equal to the half-life of seroconversion.

Open animals that were predicted to have seroconverted prior to the movement and pregnant animals and/or beef animals purchased with a calf at foot that were predicted to have seroconverted before the gestational risk period for generating a PI foetus were assumed to pose no infection risk. Pregnant animals and/or beef animals purchased with a calf at foot that were not predicted to have seroconverted prior to the gestational risk period were assigned a 3% probability of carrying a PI foetus or having a PI calf at foot, respectively, to reflect the average prevalence of PI calves at birth and the infection status was determined by a binomial trial Open animals that were predicted to be seronegative at the time of purchase were assigned a 3% probability of being transiently infected and the infection status was again determined by binomial trial. All calves born to pregnant PI dams were assumed to be PI and all animals that were transiently infected were assumed to be at the beginning of a 10 day infectious period. A total of 1000 replicates were performed for each of the 54 508 seeding events, which was deemed adequate to capture the variation in simulation outcomes based on the inspection of performance curves.

#### Disease transmission component

After the seeding event, the model was allowed to run for a maximum of 730 days or until no more infectious animals were present on the farm, (whichever occurred sooner). The outcome measure for each individual simulation run was a binary response variable of whether or not the purchased animal caused at least one additional dam in the herd to generate a PI calf over the two year period as this is an important prerequisite for disease persisting within the cattle herd [24]. The results from all 1000 replicates were then aggregated into a single variable for the seeding event, the proportion of replicates where the introduction of that animal to the herd resulted in the creation of at least one additional PI calf.

The model for the within-herd transmission dynamics of BVDV following disease introduction was adapted directly from work by Ezanno et al. [51]. Disease was assumed to spread within and between production subgroups at the frequency-dependent daily transmission rate (λ) described in Equation 3. This rate was then used in Equation 4 to calculate the probability of an individual animal acquiring BVDV (ρ_inf_) on any given day of the simulation.

3 $$ \lambda \left(g,t\right) = {\beta}_1\ \frac{P{I}_g(t)}{N_g(t)} + {\beta}_2\frac{T{I}_g(t)}{N_g(t)} + {\displaystyle \sum_{a\ \ne g}}{\beta}_3\frac{P{I}_a(t)}{N_a(t){N}_g(t)} $$

4 $$ {\rho}_{inf}\left(g,t\right)=1 - {e}^{-\lambda \left(g,t\right)} $$

Where, if *X* denotes one of the two the disease states *PI*, *TI*, then *X*_*g*_(*t*) is the number of animals in infectious state *X* within the same production subgroup, g, at time (t), *X*_*a*_(*t*) is the number of animals in infectious state *X* in all other production subgroups at time (t), β_1_ is the transmission rate from PI animals within the same production subgroup, β_2_the transmission rate from TI animals within the same production subgroup, β_3_the transmission rate from PI animals in all other production subgroups, N_g_ is equal to the total number of animals in the same production subgroup, and N_a_ is equal to the total number of animals in all other production subgroups.

**Figure 3:**
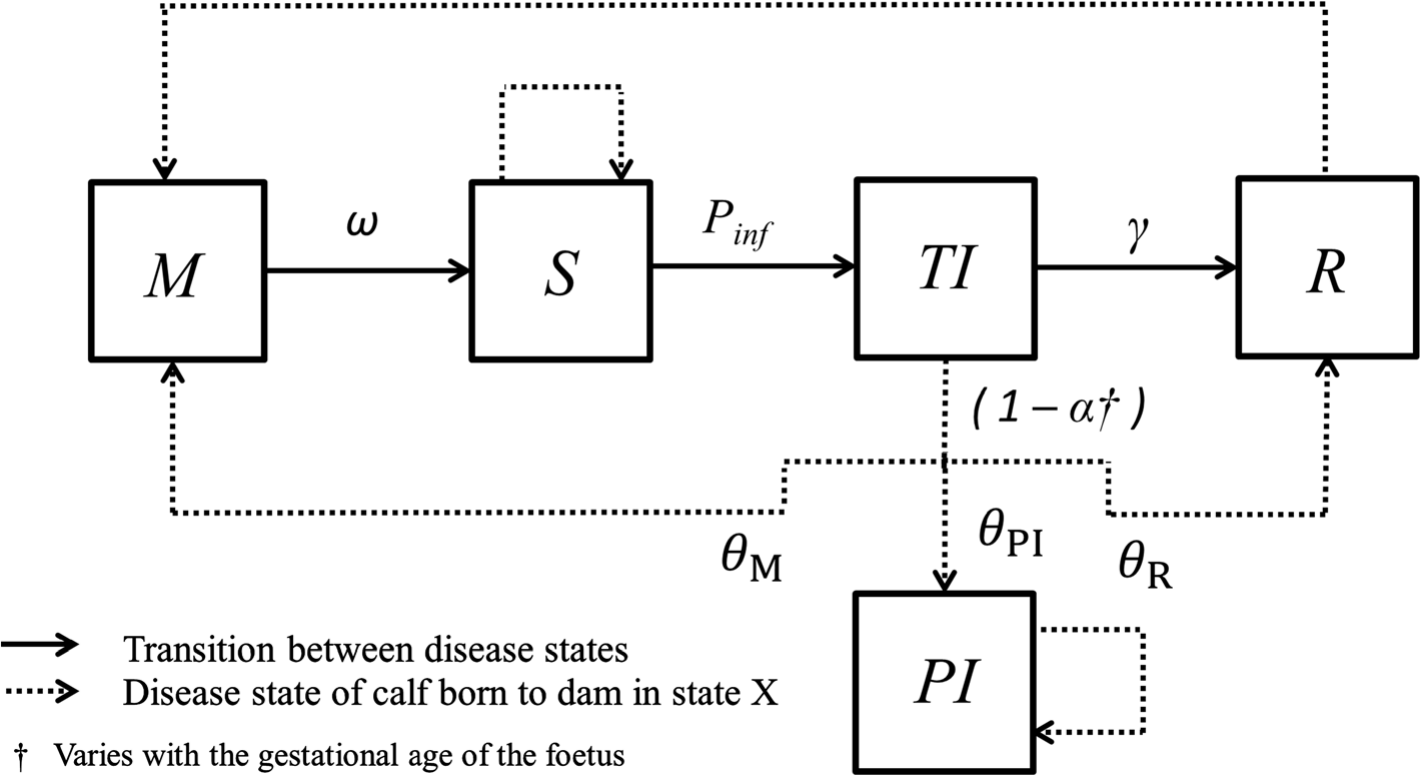
**Diagrammatic representation of the progression of animals through mutually exclusive disease states for BVDV.** This informs the disease transmission component of the within-herd BVDV simulation model. The dotted lines indicate the immunological status of calves born to dams in disease state X. The abbreviations and parameter values are defined in Table 1.

Within each production subgroup, contact with both persistently infected (PI) and transiently infected (TI) animals was assumed to lead to possible virus transmission, while between production subgroups, only PI cattle were assumed to be able to transmit virus due to their much higher viral excretion rates. The probability of an individual animal acquiring BVDV (Equation 4) was updated on each day to reflect changes in the distribution of animals across production subgroups and infection states. All horizontal transmission resulted in the movement of cattle from the susceptible (S) state to the TI state. A diagrammatic representation of the mutually exclusive infection states is shown in Figure 3 with the definition and values for the model parameters shown in Table 1.

**Table 1:** **Parameter definitions and values for the disease transmission component of the within**-**herd BVDV simulation model**

	**Definition**	**Value**	**Reference**
*M*	Calf protected by maternal antibodies	-	-
*S*	Susceptible animal	-	-
*TI*	Transiently infected animal	-	-
*PI*	Persistently infected animal	-	-
*R*	Recovered or immune animal	-	-
*ω*	Duration of maternal immunity (days)	183	[52]
*P*_inf_	Probability of infection (per day)	Eq. 2	[53]
*β*_*1*_	Within-group transmission rate from PI animals (per day)	0.5	[54]
*β*_*2*_	Within-group transmission rate from TI animals (per day)	0.03	[54]
*β*_*3*_	Between-group transmission rate from PI animals (per day)	0.1	[54]
*γ*	Recovery period for TI animals (days)	10	[52]
Early gestation (days 1 to 42)			
*α*_*e*_	Probability of abortion during early gestation	0.80	[53]
Mid-gestation (days 43 to 150) *α*			
*α*_*m*_	Probability of abortion during mid-gestation	0.25	[53]
*θ*_PI_	Probability giving birth to PI if infected during mid-gestation	0.934	[53]
*θ*_M_	Probability giving birth to M if infected during mid-gestation	0.033	[53]
*θ*_*R*_	Probability giving birth to R if infected during mid-gestation	0.033	[53]

A diagrammatic representation of the model is presented in Figure 3.

All TI cattle remained infectious for a period of 10 days (*γ*) before seroconverting and moving to the recovered (R) state. Immunity to BVD was assumed to be lifelong. For S dams infected during early gestation (days 0 to 42), there was a probability *α*_*e*_ of embryonic loss or abortion. For S dams infected during mid-gestation (days 43 to 150), there was a probability *α*_*m*_ of abortion and if the calf survived, a probability *θ*_PI_ of being PI, a probability *θ*_R_ of being R, and a probability *θ*_M_ being born protected by maternal antibodies (M). Temporary immunity to BVD through maternal antibodies was assumed to last for 183 days (ω) after which the animal joined the S group. All dams infected during early gestation (days 1 to 42) and late gestation (days 151 to 280) gave birth to M calves, all PI dams gave birth to PI calves, and all S dams gave birth to S calves. Given the short duration of the model simulation and the simple outcome measure, we assumed no increase in mortality amongst PI calves.

#### Statistical analyses

The results from the within-herd simulation models were analysed using mixed effects generalized linear models (GLM) with a binomial distribution. Data from the beef herds were analysed separately from the dairy herds due to inherent differences in management practices. The response variable was the counts of successes and failures for each seeding event and the predictor variable was the seeding event type (open heifer, pregnant heifer, open dry cow, open lactating cow, pregnant dry cow, or pregnant lactating cow). Herd was included as a random effect to account for covariance between observations on the same farm. The results from the regression model were reported as odds ratios (ORs) with the corresponding 95% confidence intervals (CIs).

### Network characteristics

Another possible explanation for the differential risk associated with the different types of replacement breeding cattle is that the source herds for these movements are also not epidemiologically equal. As there was no information on the disease status of the source herds at the time of our study, we used a simple measure from network analysis, between centrality, as an approximation of the likelihood of the source herds being infected with BVDV. Betweenness centrality measures the number of times the shortest paths between any two farms in the network pass through a particular farm [55]. In previous theoretical simulation studies, it has been shown that farms with high betweenness centrality scores have a statistically higher probability of acquiring disease and spreading disease due to their connectivity with other farms in the network [10,56]. We would therefore expect that cattle purchased from herds with a high betweenness centrality to have an increased risk of being positive for BVDV.

In our study, the individual movements of cattle in Great Britain from July 2005 through June 2006 were selected as a representative year and used to reconstruct the static movement network. For the purpose of this analysis, a farm was defined as any location classified as an agricultural holding or landless keeper (farmers housing cattle on rented land) with a Scottish county designation that housed cattle for at least one day during the study time period. For each movement, the departure location, destination location, and date were recorded. Movements that occurred through livestock markets were treated as direct movement between farms by pairing the movement onto the market location with the corresponding movement off the market location. The movements of replacement breeding cattle were identified and classified into the six production groups as previously described. The betweenness centrality scores for each source herd that supplied replacement breeding cattle to a Scottish beef or dairy herd were calculated using the igraph package for the C programming language [57]. For each production type of cattle, the median betweenness centrality of the source herds was reported.

## Results

### Empirical risk factor analysis

Only 22 of the 255 surveyed beef herds (9%) and 39 of the 189 surveyed dairy herds (21%) remained completely closed to cattle movements in the two year period prior to serological testing. This practice was associated with a significantly decreased odds of being seropositive for BVDV (OR: 0.12, 95% CI: 0.01 – 0.60, *p* = 0.041 for beef herds and OR: 0.38, 95% CI: 0.18 – 0.78, *p* = 0.008 for dairy herds). Amongst the open herds, beef herds that purchased replacement breeding cattle had a 2.09 times greater odds of being seropositive for BVD (95% CI: 1.06 – 4.39, *p* = 0.040) compared with herds that purchased store cattle only. Similarly, open dairy herds that purchased replacement breeding cattle were 2.67 times more to be seropositive for BVDV (95% CI: 1.32 – 5.52, *p* = 0.006) than open herds that purchased store cattle only.

Results from the empirical risk factor analysis predicted that the risk of a herd being seropositive for BVDV varied based on the types of replacement breeding cattle purchased during the two year period prior to serological testing. Open beef herds that purchased pregnant heifers, open cows with a calf at foot and pregnant dry cows were at significantly increased risk of being seropositive for BVDV in the univariable analyses (Table 2). In the multivariable model, only the former two variables remained significant. The odds of a beef herd being seropositive for BVDV were 2.18 times greater with the purchase of pregnant heifers (95% CI: 1.17 – 4.08, *p* = 0.014) and 2.09 times greater with the purchase of open cows with a calf at foot (95% CI: 1.13 – 3.88, *p* = 0.018). For open dairy herds, the odds of being seropositive for BVDV increased with the purchase of open heifers, open dry cows, and open lactating cows in the univariable analysis (Table 2). However, when combined in the multivariate model, only the purchase of open heifers remained a significant predictor at the 0.05 level (OR 3.06, 95% CI: 1.46 – 6.77, *p* = 0.004).

**Table 2:** **Univariable analysis of risks for BVDV seropositivity associated with different types of beef and dairy replacement breeding cattle movements**

	**Number of herds with movements**		**Number of herds without movements**				
**Type of cattle movements**	**Cases**	**Controls**	**Cases**	**Controls**	**OR**	**95% CI**	***p*** **-value**
Beef herds							
Open heifers	40	83	26	84	1.56	0.88 – 2.81	0.134
Pregnant heifers	32	43	34	124	2.74	1.50 – 4.94	< 0.001
Open cows (dry)	16	26	50	141	1.73	0.85 – 1.74	0.123
Open cows (calf at foot)	37	55	29	112	2.60	1.45 – 4.69	0.001
Pregnant cows (dry)	28	32	38	135	3.11	1.67 – 5.81	< 0.001
Pregnant cows (calf at foot)	17	38	49	129	1.18	0.59 – 2.25	0.627
Dairy herds							
Open heifers	54	12	50	34	3.06	1.46 – 6.77	0.004
Pregnant heifers	43	14	61	32	1.61	0.78 – 3.45	0.206
Open cows (dry)	22	3	82	43	3.84	1.24 – 16.9	0.036
Open cows (lactating)	50	13	54	33	2.35	1.13 – 5.10	0.025
Pregnant cows (dry)	22	7	82	39	1.49	0.61 – 4.05	0.398
Pregnant cows (lactating)	26	7	78	39	1.86	0.77 – 4.98	0.187

Records of all cattle movements into the 233 open Scottish beef herds and 150 open Scottish dairy herds during the two year period prior to the date of serological sampling were used to generate the binary categorical movement variables listed in the table below.

### Within-herd simulation model

Results from the within-herd simulation models are presented in Table 3. Compared with the movements of open beef heifers, the movements of pregnant beef heifers had 1.76 times greater odds (95% CI: 1.74 – 1.77, *p* < 0.001) of generating additional PI calves and the movements of open beef cows with a calf at foot had 1.23 times greater odds (95% CI: 1.22 – 1.24, *p* < 0.001) despite both groups having a lower probability of being PI cattle based on their age at the time of movement. For dairy herds, the movements of open heifers had the greatest odds of generating additional PI calves compared with all other animal production types. The movements of open dry cows had the lowest risk compared to the movements of open heifers for both beef and dairy herds, although these movements were relatively infrequent.

**Table 3:** **Simulated risk of generating additional PI calves associated with cattle purchased by open beef and dairy herds**

	**Number of seeding events**	**Average age related probability of being PI (×10** ^**−2**^ **)**	**% simulation runs resulting in outbreak** ^**†**^	**OR**	**95% CI**
Beef herds					
Open heifers	22 473	1.37	1.32	Ref	-
Pregnant heifers	5205	0.63	2.32	1.76	1.74 – 1.77
Open cows (dry)	1184	0.16	0.16	0.13	0.12 – 0.13
Open cows (calf at foot)	8269	0.28	1.73	1.23	1.22 – 1.24
Pregnant cows (dry)	5131	0.09	0.82	0.68	0.67 – 0.79
Pregnant cows (calf at foot)	2176	0.15	1.19	0.94	0.92 – 0.95
Dairy herds					
Open heifers	3188	0.84	2.00	Ref	-
Pregnant heifers	1734	0.54	1.95	0.92	0.90 – 0.93
Open cows (dry)	275	0.11	0.86	0.44	0.42 – 0.46
Open cows (lactating)	3510	0.30	1.79	0.67	0.66 – 0.68
Pregnant cows (dry)	547	0.10	1.20	0.64	0.62 – 0.66
Pregnant cows (lactating)	769	0.16	1.49	0.68	0.67 – 0.70

^†^The percentage of simulation replicates that resulted in the generation of at least one additional PI calf in the herd within 2 years of the original seeding event.

The results are based on a dynamic within-herd simulation model that makes use of the explicit demographic structure and movement patterns of 2895 beef herds and 546 dairy herds in Scotland from records stored in the Cattle Tracing System (CTS) database. A total of 1000 replicates were performed for each disease seeding event in the herds to capture the stochastic variation in the probability of purchased replacement breeding cattle being persistently or transiently infected with BVDV and the probability of generating at least one additional PI calf in the herd following disease introduction. The odds of particular types of replacement breeding cattle causing outbreaks were expressed relative to the “open heifer” group, which had the highest probability of being PI based on the average animal age at time of purchase.

### Network characterizations

There were a total of 4934 Scottish beef herds and 804 Scottish dairy herds that sold replacement breeding cattle from 01 July 2005 through 30 June 2006. The median source herd betweenness centrality scores for herds that supplied each type of replacement breeding cattle is shown in Table 4. For beef replacement cattle, the median source herd betweenness centrality scores for open cows with calf at foot (199 017) and pregnant heifers (106 630) were appreciably higher than for the other production groups (range: 66 177 to 82 251). For dairy replacement cattle, the highest median betweenness centrality score of source herds was observed for open lactating cows (192 613) and open heifers (137, 442), while the median source herds betweenness centrality scores for the other production groups ranged from 109 568 to 129 386.

**Table 4:** **Source herd betweenness centrality scores for beef and dairy replacement breeding cattle purchased in Scotland**

	**Number of replacement breeding cattle**	**Number of herds**	**Median source herd betweenness centrality**
Beef replacement cattle			
Open heifers	16 420	3229	82 521
Pregnant heifers	5795	1369	106 630
Open cows (dry)	1150	565	74 212
Open cows (calf at foot)	8458	1348	199 017
Pregnant cows (dry)	8491	1226	66 177
Pregnant cows (calf at foot)	3900	745	70 871
Dairy replacement cattle			
Open heifers	2916	430	137 442
Pregnant heifers	2643	352	110 976
Open cows (dry)	707	223	111 983
Open cows (lactating)	3839	538	192 613
Pregnant cows (dry)	1276	230	109 568
Pregnant cows (lactating)	1330	217	129 386

Data from 01 July 2005 through 30 June 2006 is reported. There were a total of 4934 beef herds and 804 dairy herds that sold replacement breeding cattle. Note that some herds sold multiple types of replacement breeding cattle.

### Results summary

The predicted risks associated with the different types of replacement breeding cattle movements across the three study analyses are summarized in Table 5. Overall, open beef cows with a calf at foot, pregnant beef heifers, and open dairy heifers were associated with the highest potential risk of causing BVDV outbreaks through cattle movements.

**Table 5:** **Summary of the predicted relative risks associated with different types of beef and dairy replacement breeding cattle movements**

	**Empirical risk factor analysis** ^**†**^	**Within-herd simulation model** ^**‡**^	Network characterizations*	**Overall risk score**
Beef replacement cattle				
Open cows (calf at foot)	2.09	1.23	2.41	5.73
Pregnant heifers	2.18	1.76	1.29	5.23
Pregnant cows (calf at foot)	1.00	1.19	0.86	3.05
Open heifers	1.00	1.00	1.00	3.00
Pregnant cows (dry)	1.00	0.82	0.80	2.62
Open cows (dry)	1.00	0.16	0.90	2.06
Dairy replacement cattle				
Open heifers	3.06	1.00	1.00	5.06
Open cows (lactating)	1.00	0.67	1.40	3.07
Pregnant heifers	1.00	0.92	0.81	2.73
Pregnant cows (lactating)	1.00	0.68	0.94	2.62
Pregnant cows (dry)	1.00	0.64	0.80	2.44
Open cows (dry)	1.00	0.44	0.81	2.25

^†^Based on the odds ratios from the multivariable analysis. Replacement breeding cattle groups that were not significant in the multivariable analysis were assigned a score of 1.00.

^‡^Based on the odds ratios directly reported in the original within-herd simulation model analysis.

*Calculated as the magnitude difference in betweenness centrality scores relative to the value for open heifers.

An overall risk score was calculated for each replacement breeding cattle group by summing the relative risks predicted by each of the study analyses.

## Discussion

Similar to other empirical risk factor analyses [31,32,58], there was strong evidence that cattle movements in general were associated with an increased risk of herds being seropositive for BVDV. However, we also found that the risk varies based on the animal and herd demographic characteristics at the time of purchase. For beef herds, pregnant heifers and open cows with a calf at foot were identified as high risk groups in the multivariable model, while for dairy herds, open heifers appeared to have the greatest risk. These results are in accordance with anecdotal evidence from throughout the world (Gunn, G.J. pers. comm. 2014) and therefore provide valuable empirical evidence in their favour. While it is possible that these findings may simply be an artefact of the small sample size or may simply be proxies for other management practices associated with BVDV transmission, results from the within-herd simulation model and the network characterizations provide additional theoretical support to explain the differences in predicted risk.

The within-herd simulation model used published estimates from the literature to determine the age-related probability of purchased cattle being persistently infected, transiently infected, or immune from previous exposure to BVDV. By using movement records from the CTS database to characterize herd demographic structures, the models also explicitly accounted for the probability of purchased cattle being introduced to the herd when susceptible dams were in the gestational risk period for generating additional PI calves. To our knowledge, only one other published study to date has used CTS records in this manner [13] and this represents a significant advance in developing more realistic models of BVDV transmission dynamics. We recognize that the parameter values used to model within-herd transmission dynamics are assumed constant whereas in reality they may vary between herd types. Therefore the magnitude of the results must be interpreted with some caution.

Pregnant replacement breeding cattle are a known epidemiological control challenge for beef herds since there are few reliable prenatal diagnostic tests to identify animals carrying a PI calf [38-40] and once born, the calves mix directly with susceptible breeding dams until weaning at approximately 6 to 8 months of age. Given that the majority of farmers in Scotland do not routinely screen cattle at the time of purchase [59], it is unlikely that calves are tested after birth to identify and remove PIs [60,61]. Similar concerns are present for dams that are purchased with a calf at foot, especially if they are introduced to the herd at the start of the breeding season when a large number of dams are in the gestational risk period for generating PI calves. We would expect that older dams are less likely to be PI and more likely to be immune from previous exposure, which may partly explain why the purchase of pregnant cows with a calf at foot was not a significant risk factor for seropositivity in beef herds. Interestingly, the purchase of any type of pregnant cattle was not a significant risk factor for BVDV seropositivity in dairy herds. One possible explanation is that dairy calves are removed from their dams within 24 h of birth and either sold directly to fattening units for beef production or raised in separate production units on the farm. Therefore, even if a pregnant replacement dam gives birth to a PI calf, there are fewer opportunities for disease to spread to susceptible breeding cattle. There may also be underlying differences in the management practices and within-herd transmission dynamics of source herds supplying pregnant replacement dairy cattle that reduce the risk of these animals carrying PI calves.

In contrast, the purchase of open heifers and open breeding cattle in general was a significant risk factor for seropositivity in dairy herds, but not for beef herds. One possible explanation is that beef heifers were purchased at a much younger age than dairy heifers and under certain circumstances, it has been shown that exposing susceptible heifers to PI cattle can be beneficial in inducing protective immunity to prevent future transient BVDV infections during early gestation [52]. Most open dairy heifers were introduced to the herd closer to the average age at first calving for the dairy industry and may have been more likely to be commingled with susceptible breeding cattle. Based on findings from the within-herd simulation model, there are also likely differences in the herd demographic structure (i.e. percentage of dams in the gestational risk period for generating PI calves) at the time when these particular types of replacement breeding cattle were introduced that influence the risk of BVDV subsequently spreading through the herd. A previous investigation into the management practices of the herds examined in our study showed significant differences in the basic level of biosecurity that beef and dairy herds adopted for purchased cattle [59]. However, the survey questions were simple binary response variables and it was not possible to assess whether the biosecurity measures were applied differently across the various types of replacement breeding cattle. This highlights the need for more basic research into understanding how these heterogeneities in herd management impact the both the within-herd and between-herd transmission dynamics of infectious cattle diseases.

In this interpretation, we also assumed that replacement breeding cattle movements were a cause of BVDV infections rather than a consequence. Herds that are actively infected with BVDV may need to purchase additional replacement breeding animals to compensate for the negative effects on fertility, reproductive performance, and mortality (due to mucosal disease and immunosuppression) [62-64]. As highlighted by Lindberg and Alenius [65], purchasing susceptible breeding replacement cattle may contribute to the persistence of BVDV in these herds if these animals become infected during gestation. This could be an important consideration for national control programmes that focus primarily on preventing infected animals from entering susceptible breeding herds.

There was also evidence that our findings may be related to the differing epidemiological risk associated with herds that sell particular types of replacement breeding cattle. Herds that sold pregnant replacement beef heifers and open beef cows with a calf at foot had higher median betweenness centrality scores than herds that sold other types of cattle, which may indicate an increased likelihood that they were infected with BVDV. In short it is possible that it is not the type of replacement animal *per se* that matters, but could be the connectedness of herds to others that tend to transfer that particular type of animal. A recent study from Sweden found that herds with large ingoing infection chains (another measure which accounts for both the number and connectivity of source herds for purchased cattle) were significantly more likely to be seropositive for bovine coronavirus and bovine respiratory syncytial virus than more isolated herds [66]. With the limitations of the small study sample, it was not possible to investigate whether herds that sold replacement breeding cattle were more likely to be seropositive for BVDV and whether placing increased biosecurity restrictions on herds that sell replacement breeding cattle would be a more cost-effective control strategy. These questions may be answered in the near future as more comprehensive surveillance data from the recently launched Scottish BVD Eradication Scheme becomes available [67]. We also did not assess the potential for BVDV to spread between cattle at livestock market sales, which may be another important mechanism of disease transmission in the Scottish cattle industry.

Any test which seeks to detect an antibody that arises from historic infection, present infection, vaccination and introduction of an antibody positive animal is likely to be an imperfect test. This applies to the testing for BVDV antibodies in bulk milk and interpretation of the test is made more confused by the fact that currently most dairy herds in Scotland buy in replacement heifers. Such factors probably explain our observation that the trends in movement and network risks associated with purchased replacement breeding cattle were less clear for dairy herds than for beef herds [68,69]. Although we intentionally excluded herds that reported using BVDV vaccines, we recognize that the simple question of “Do you routinely vaccinate your herd for BVD?” does not allow for variation in the number of cattle vaccinated, the use of different vaccination strategies for different management groups, and the past use of vaccinations in the breeding herd, all of which may result in the presence of antibody positive cattle in the absence of an active BVDV infection. In a previously published analysis of these data [59], we also found evidence that local transmission mechanisms may be relatively more important for dairy herds than for beef herds, which may further weaken the associations between recent cattle movements and BVDV seropositivity in dairy herds.

There were also limitations in how we used the CTS data to define animal production types and epidemiologically relevant cattle movements. For example, it was not possible to distinguish heifers that were purchased as store cattle for fattening from those that were purchased as replacement breeding animals, but culled prior to calving. This may have underestimated the number of herds that purchased replacement breeding cattle as well as the potential risk associated with purchasing open heifers. Additional bias may have been introduced to the empirical risk factor analysis through the exclusion of farms that could be linked to a valid CPH code through their reported address. However, we believe this is unlikely to have impacted the main study findings since there is no obvious reason why the unmatched farms would have a higher prevalence of BVDV. This highlights the importance of recording accurate farm identification information in future epidemiological studies.

The primary motivation behind this study was to emphasize that not all cattle movements and not all cattle herds are epidemiologically equal. Although BVDV was used as a case example, the basic principles and methodologies are equally relevant to the many other infectious cattle diseases that spread through cattle movement networks, such as bovine tuberculosis, bovine paratuberculosis, bovine herpesvirus 1, bovine leukaemia virus, and contagious mastitis. Most importantly, this analysis demonstrates how the basic records of individual births, deaths, and movements available through national cattle movement databases can be used in future research studies to better quantify disease specific risk factors for infectious diseases and to determine the relative importance of high risk movements to disease transmission dynamics at the population level.
